# Neutrophil TLR4 expression is reduced in the airways of infants with severe bronchiolitis

**DOI:** 10.1136/thx.2008.107821

**Published:** 2009-06-03

**Authors:** C P Halfhide, S P Brearey, B F Flanagan, J A Hunt, D Howarth, J Cummerson, S Edwards, C A Hart, R L Smyth

**Affiliations:** 1 Division of Child Health, School of Reproductive and Developmental Medicine, University of Liverpool, Alder Hey Children’s Hospital, Liverpool, UK; 2 Division of Immunology, University of Liverpool, Liverpool, UK; 3 Division of Clinical Engineering, University of Liverpool, Liverpool, UK; 4 Division of Biological Sciences, University of Liverpool, Liverpool, UK; 5 Division of Medical Microbiology, University of Liverpool, Liverpool, UK

## Abstract

**Background:**

In respiratory syncytial virus (RSV) bronchiolitis, neutrophils account for >80% of cells recovered from the airways in bronchoalveolar lavage (BAL) fluid. This study investigated neutrophil activation and Toll-like receptor (TLR) expression in the blood and lungs of infants with severe RSV bronchiolitis.

**Methods:**

BAL fluid and (blood) samples were collected from 24 (16) preterm and 23 (15) term infants ventilated with RSV bronchiolitis, and 12 (8) control infants. Protein levels and mRNA expression of CD11b, myeloperoxidase (MPO) and TLRs 2, 4, 7, 8 and 9 were measured in neutrophils.

**Results:**

Blood neutrophils had more CD11b in preterm and term infants with RSV bronchiolitis than control infants (p<0.025) but similar amounts of MPO. BAL fluid neutrophils from infants with RSV bronchiolitis had greater amounts of CD11b and MPO than blood neutrophils and BAL fluid neutrophils from controls (p<0.01). Blood neutrophils from term infants with RSV bronchiolitis had less total TLR4 protein than preterm infants with RSV bronchiolitis (p = 0.005), and both had less than controls (p<0.04). Total TLR4 for each group was greater in BAL fluid neutrophils than in blood neutrophils. Blood neutrophils from preterm infants with RSV bronchiolitis had greater TLR4 mRNA expression than term infants with RSV bronchiolitis (p = 0.005) who had similar expression to controls (p = 0.625).

**Conclusions:**

In infants with severe RSV bronchiolitis, neutrophil activation starts in the blood and progresses as they are recruited into the airways. Total neutrophil TLR4 remains low in both compartments. TLR4 mRNA expression is unimpaired. This suggests that neutrophil TLR4 expression is deficient in these infants, which may explain why they develop severe RSV bronchiolitis.

Respiratory syncytial virus (RSV) bronchiolitis is the commonest cause of lower respiratory tract infection in children under the age of 1 year.^1^ Symptoms begin in the upper airways and it is likely that the primary site of RSV infection is the nasal epithelium^2^ with local spread to the lower airways.^3^ Initial infection of the airways leads to production of pro-inflammatory cytokines^4^ ^5^ and chemokines^6^ which initiates the recruitment of inflammatory cells from the peripheral circulation. There is little evidence that RSV causes a viraemia,^7^ but the immunopathogenesis of RSV bronchiolitis is poorly understood. By studying cells from the blood and airways of infants with RSV bronchiolitis and comparing them with uninfected controls, information can be elucidated about the changes that inflammatory cells undergo as they are recruited to the airways.

The clinical spectrum of RSV infection is wide, from a mild upper respiratory tract infection to severe lower respiratory tract infection.^1^ Risk factors for severe RSV bronchiolitis are prematurity (particularly with associated chronic lung disease),^8^ congenital heart disease^9^ and immunodeficiency.^10^ Half of infants ventilated for RSV bronchiolitis are born at term with no risk factors.^1^ ^11^ In previous studies we have consistently shown that term infants have a more vigorous immune response to RSV infection in their airways than preterm infants,^4^ ^5^ ^12^ and this may relate to the considerable maturation that occurs in the third trimester and in the first year of life.^13^ ^14^

Neutrophils are the predominant cell found in the inflammatory infiltrate of the bronchoalveolar lavage (BAL) fluid of infants ventilated for severe RSV bronchiolitis.^5^ They are recruited to the lungs early in the course of infection where they are known to release cytokines^12^ and show delayed apoptosis.^15^ ^16^ As the clinical condition improves, lower neutrophil concentrations are found in the BAL fluid.^4^ ^5^ Neutrophil activation can be studied by looking at cell surface markers such as the integrins CD11b or internal markers such as myeloperoxidase (MPO) found in the neutrophil granules. Integrin expression is quickly upregulated by mobilisation of internal stores. MPO is released to the cell surface when the neutrophil is degranulating at the site of active inflammation.

Toll-like receptors (TLRs) recognise viral and bacterial pathogen-associated molecular patterns and initiate a specific inflammatory response to a range of infections.^17^ In RSV infection, TLRs may play an important role in regulating innate and adaptive immune responses.^18^ ^19^ ^20^ Immune responses mature with gestation and during the first few months of life when infants encounter RSV infection.^21^

Circulating neutrophils express all human TLRs except TLR3,^22^ but the contribution of TLRs to the pathogenesis of RSV disease is not fully understood. There has been considerable interest in the role of TLR4 in regulating RSV infection since RSV F protein has been reported to be a ligand for TLR4.^23^ A clinical study found increased TLR4 expression in blood monocytes of infants with RSV bronchiolitis,^24^ and a genetic susceptibility study identified two common TLR4 gene mutations which are associated with an increased risk of severe RSV bronchiolitis compared with mild disease (odds ratio 5.1 (95% confidence interval (CI) 1.4 to 18.1) and 4.0 (95% CI 1.3 to 12.5).^25^

Intracellular TLRs (3, 7, 8 and 9) recognise viral and bacterial nucleic acid in the endosomes of infected cells. RSV has been shown to interfere with TLR7 and TLR9 signalling pathways in plasmacytoid dendritic cells, resulting in significant reductions in type I interferon production.^20^ RSV may also induce cytokine secretion and mucus production in airway epithelial cells via TLR3 signalling.^26^

We have undertaken a large study to investigate neutrophil activation and TLR expression in the blood and BAL fluid of infants with severe RSV bronchiolitis (term and preterm) compared with control infants to delineate what changes occur in neutrophils as they migrate to the lungs from the systemic circulation.

## Methods

### Study population and sample collection

Forty-seven consecutive infants aged <1 year admitted with severe RSV bronchiolitis to the intensive care unit at Royal Liverpool Children’s Hospital, Alder Hey over three winter seasons were recruited to the study. RSV bronchiolitis was confirmed by direct immunofluorescence of nasopharyngeal aspirates. After informed consent, samples of peripheral blood and BAL fluid were collected as previously described.^4^ ^5^ ^12^ We recruited 24 participants who were born prematurely (<37 weeks gestation) and 23 who were born at term (⩾37 weeks gestation). Data from infants born at term were analysed separately from those infants born preterm. Infants with underlying cardiorespiratory disease were excluded. The control group comprised 12 uninfected healthy infants of the same age as the infants with RSV bronchiolitis who had been ventilated prior to elective non-cardiac surgery, from whom blood and BAL fluid samples were collected immediately after induction of anaesthesia and intubation of the airway. No recruited infant was subsequently withdrawn from the study. All samples were processed and data analysed from each recruited infant. During the first year of the study we collected BAL fluid but not blood samples.

#### Preparation of BAL fluid samples

Standard techniques were used to assess total and differential cell counts.^8^ ^9^ ^16^ ^27^ Briefly, BAL fluid was filtered (60 μm pore size gauze; Sefar Nitex 03-48/31, Sefar, Switzerland) to remove bulk mucus and then centrifuged. The supernatant was removed and stored at −70°C. The cellular component was resuspended in RPMI 1640 and the neutrophils purified using sedimentation gradient techniques on PolymorphPrep (Axis-Shield PoC AS, Norway). We took 59 BAL fluid samples, 47 from infants with RSV bronchiolitis (24 preterm and 23 term infants) and 12 from healthy control infants. The number of cells was highest in those infants with RSV bronchiolitis born at term and, as in previous studies,^4^ ^5^ ^12^ neutrophils were the predominant cell (table 1). After enrichment, >93% of the cells were neutrophils with >94% viability; the median (SD) percentages of other cells were macrophages 4 (3)%, lymphocytes 2 (1)%.

**Table 1:** Characteristics of patients and bronchoalveolar lavage (BAL) fluid and blood samples before enrichment

	Infants with bronchiolitis		Control infants
	Preterm	Term	
*Clinical information*			
No of patients	24	23	12
Median (range) age on admission (weeks)	3.5 (−5 to 11.7)	16 (4–56)	12 (6–20)
Weight on admission (kg)	3.3 (0.35)	5.1 (0.36)	4.3 (2.0)
Median (range) gestation at birth (weeks)	34 (27–36)	40 (39–42)	40 (37–40)
*BAL fluid cell counts*			
Total cell concentration (×10^6^ cells/ml)	1.8 (0.8)	1.9 (0.5)	1.5 (0.8)
Total cell count (×10^6^)	4.5 (1)	4.8 (1.2)	3 (0.5)
Percentage of viable cells	95 (2.5)	94 (2)	95 (1)
Median (range) percentage neutrophils	84 (2.5)	83 (2)	37 (3)
Median (range) percentage alveolar macrophages	12.5 (1.5)	11.6 (1.0)	47 (3)
Median (range) percentage lymphocytes	5 (0.5)	3.5 (1.0)	7 (3)
*Blood cell counts*			
No of patients	16	15	8
Total cell concentration (×10^9^ cells/l)	9.95 (1.67)	9.95 (1.64)	9.44 (1.5)
Percentage of viable cells	97 (2)	96 (1)	96 (1)
Mean concentration (×10^9^/l neutrophils)	4.93 (1.46)	5.11 (1.48)	4.5 (1.56)
Mean concentration (×10^9^/l monocytes)	0.99 (0.15)	1.11 (0.17)	1.0 (0.18)
Mean concentration (×10^9^/l lymphocytes)	3.67 (0.3)	3.57 (0.44)	3.63 (0.35)
Mean concentration (×10^9^/l eosinophils)	0.088 (0.04)	0.078 (0.029)	0.082 (0.045)

Data mean (SEM) unless stated otherwise.

#### Preparation of blood neutrophils

Neutrophils were purified from whole blood by sedimentation gradient as described above. Thirty-nine blood samples were taken, 31 from infants with RSV bronchiolitis (16 preterm and 15 term) and 8 from healthy control infants. There was no difference in the total cell count between cases and control infants (table 1). After enrichment, >95% of the cells were neutrophils with >96% viability; the median (SD) percentages of other cells were monocytes 2 (1)%, lymphocytes 2 (1)%.

### FACS analysis: protein expression of CD11b, MPO and TLRs 2, 4, 7, 8 and 9

All cells for FACS analysis were triple stained with ethidium monoazide bromide (EMA) (Biotium, UK) to allow exclusion of non-viable cells, a neutrophil marker (MPO or CD16) and the test antibody (TLRs 2, 4, 7, 8, 9, CD11b, MPO) or isotype control.

For cell surface expression of CD11b, TLR2 and TLR4 neutrophils were labelled and fixed (BD cytofix: 1% paraformaldehyde).

For total expression of CD11b, MPO and TLRs 2, 4, 7, 8 and 9 neutrophils were permeabilised, fixed (BD Cytofix and Cytoperm; BD Biosciences, Oxford, UK) and labelled. Samples were analysed on a FACSort flow cytometer (Becton Dickinson) collecting a minimum of 10 000 events. Data were processed using WinMDI Version 2.8. Neutrophils were gated by size, granularity, viability and CD16 or MPO expression. TLR expression was standardised from the mean fluorescent intensity (MFI) into antigen binding capacities (ABC) relative units^28^ to allow quantification of the antigen binding sites between different commercial antibodies, each with different binding affinities, according to the manufacturer’s instructions (Simple Cellular Antigen-Bangs Laboratories, Kidlington, UK) (table 2).

**Table 2:** Antibodies used for flow cytometry

	Antibody type	Manufacturer
Antibody raised against		
TLR2	FITC mouse monoclonal IgG_2a_	Imgenex
TLR4	FITC mouse monoclonal IgG_2a_	
TLR7	FITC mouse monoclonal IgG_1_	
TLR8	FITC mouse monoclonal IgG_1_	
TLR9	FITC mouse monoclonal IgG_1_	
CD16	PE mouse monoclonal IgG_1_	BD BioSciences
MPO	PE mouse monoclonal IgG_1_	DakoCytomation
CD11b	FITC mouse monoclonal IgG_1_	BD Biosciences
Isotype antibodies		
	FITC/PE mouse IgG_1_ control	Imgenex
	FITC/PE mouse IgG_2a_ control	

MPO, myeloperoxidase.

### Real-time polymerase chain reaction (RT-PCR)

RNA was prepared from 0.5×10^6^ neutrophils using Trizol (Gibco, Basingstoke, UK), 5 μg of DNAse-treated total RNA was used as a template for first strand cDNA synthesis in a 20 μl reaction using Multiscribe reverse transcriptase (Applied Biosystems, Foster City, California, USA).

The mRNA expression of TLRs 2, 4, 7, 8 and 9 was assessed using Taqman primer/probe sets (Applied Biosystems). All PCR reactions were in triplicate using an Applied Biosystems 7300 and standardised to the housekeeping gene L32 using the ΔCt method.

### Statistical analysis

Protein expression of CD11b, MPO and protein and mRNA expression of TLRs 2, 4, 7, 8 and 9 were compared from enriched neutrophil blood and BAL fluid samples in preterm and term infants with RSV bronchiolitis and controls. Differences in means between patient groups were examined using one-way analysis of variance and independent sample *t* tests. As paired data were not available on all infants for comparison of the same neutrophils in blood and BAL fluid, pairing was ignored leading to more conservative analyses, but an analysis using only paired data is available in table 3 in the online supplement. Data were analysed using SPSS Version 15 (SPSS, Chicago, Illinois, USA). A probability level of 95% (p<0.05) was considered as the threshold for statistical significance.

## Results

### Protein expression of neutrophil activation markers in infants with RSV disease

**Figure 1:**
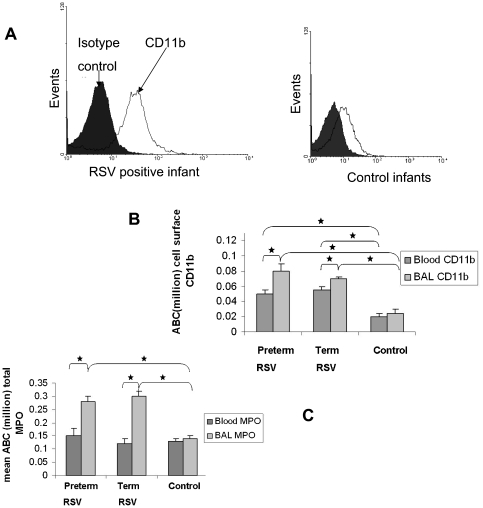
Protein expression of neutrophil activation markers is upregulated in respiratory syncytial virus (RSV) disease. Data are presented as mean (SEM) arbitrary units of antigen binding capacity (ABC) ×10^6^. (A) Histograms of flow cytometry data from two representative patients, an infant with RSV bronchiolitis (left) and a control infant (right), showing the mean protein expression of CD11b in the bronchoalveolar lavage (BAL) fluid compared with isotype control (shaded) (B) To determine mean cell surface CD11b expression, blood and airway neutrophils were stained, fixed and analysed by flow cytometry. CD11b expression was similar for term (n = 15) and preterm infants (n = 16) with RSV bronchiolitis, and both were significantly greater than controls (n = 8). There was significantly greater cell surface CD11b protein expression in the BAL neutrophils than in the blood neutrophils. (C) To determine mean total myeloperoxidase (MPO) expression, neutrophils from blood and BAL fluid were fixed and permeabilised before staining and analysed by flow cytometry. For blood neutrophils there was no difference in protein expression of MPO between infants with bronchiolitis (n = 31) and controls (n = 8). For the BAL fluid neutrophils, both term (n = 15) and preterm infants (n = 16) with RSV bronchiolitis had significantly more MPO expression than control infants (n = 8). There was significantly more MPO in BAL fluid than in blood. *p<0.05.

All neutrophils in the blood and BAL fluid were positive for CD11b and MPO expression. All data presented are expressed as mean (SEM) relative ABC units (×10^6^). The amounts of CD11b in blood neutrophils from both preterm and term infants with RSV bronchiolitis (0.05 (0.005) and 0.055 (0.005), respectively) were greater than in control infants (0.02 (0.005), p<0.025). The amounts of CD11b in BAL fluid neutrophils in preterm and term infants with RSV bronchiolitis (0.08 (0.001) and 0.07 (0.002), respectively) were greater than in controls (0.025 (0.005), p<0.012; fig 1A and B).

There was no difference between preterm and term infants with RSV bronchiolitis and controls in the amount of blood neutrophil MPO (0.15 (0.03), 0.12 (0.02) and 0.13 (0.01), respectively; p>0.05). The amount of MPO was greater in BAL fluid neutrophils from both preterm and term infants with RSV bronchiolitis (0.28 (0.02) and 0.30 (0.02), respectively) than from control infants (0.14 (0.01); p<0.05; fig 1C). There were no significant differences in the expression of CD11b or MPO between preterm and term infants in blood or BAL fluid neutrophils. For both groups of infants with severe RSV bronchiolitis, there were greater amounts of CD11b and MPO in neutrophils from the BAL fluid than from the blood. There was no difference between these compartments for either marker in control infants.

### Expression of TLRs in infants with RSV bronchiolitis and controls

**Figure 2:**
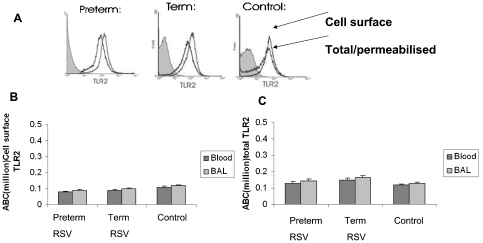
Toll-like receptor (TLR) 2 protein expression is unaltered in infants with severe respiratory syncytial virus (RSV) bronchiolitis. All data presented as mean (SEM) arbitrary units of antigen binding capacity (ABC) ×10^6^. (A) Representative histograms from flow cytometric staining of TLR2 on bronchoalveolar lavage (BAL) fluid neutrophils from three infants, a preterm infant with RSV bronchiolitis, a term infant with RSV bronchiolitis and a control infant. (B) The mean expression of cell surface TLR2 was determined by staining and fixing neutrophils prior to analysis by flow cytometry. The mean expression of cell surface TLR2 was similar between all three patient groups for airway and blood neutrophils (preterm infants, n = 16; term infants, n = 15; controls, n = 8) and for each group between blood and BAL fluid neutrophils. (C) To determine total TLR2, the neutrophils were fixed and permeabilised prior to analysis by flow cytometry. Total neutrophil TLR2 was similar between all three patient groups in both blood and BAL fluid (preterm RSV, n = 16; term RSV, n = 15; control, n = 8) and between blood and BAL fluid for each group.

**Figure 3:**
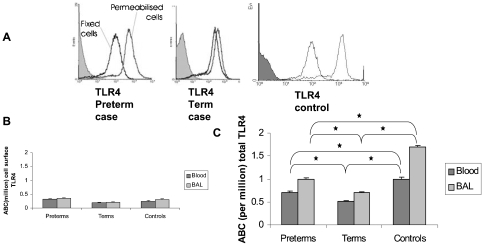
Toll-like receptor (TLR) 4 protein expression is downregulated in infants with severe respiratory syncytial virus (RSV) bronchiolitis. Data presented as mean (SEM) arbitrary units of antigen binding capacity (ABC) ×10^6^. (A) Representative histograms from flow cytometric staining of TLR4 on bronchoalveolar lavage (BAL) fluid neutrophils from three infants, a preterm and a term infant with RSV bronchiolitis and a control infant. TLR4 expression was greater than TLR2. For TLR4, term infants with bronchiolitis had less total TLR4 than preterm infants with bronchiolitis and control infants. Shaded histograms are isotype controls. (B) Expression of cell surface TLR4 was analysed by flow cytometry after staining and fixing neutrophils. There was no significant difference between patient groups for cell surface expression of neutrophil TLR4 for either BAL fluid neutrophils (preterm RSV, n = 24; term RSV, n = 23; controls, n = 12) or blood neutrophils (preterm RSV, n = 16; term RSV, n = 15; controls, n = 8) or between blood and BAL fluid neutrophils for each patient group. (C) To determine total neutrophil TLR4 expression, cells were fixed and permeabilised before analysis by flow cytometry. In both blood and BAL fluid neutrophils, term and preterm infants with RSV bronchiolitis had significantly less TLR4 expression than control infants. Term infants with RSV bronchiolitis had significantly less total TLR4 than preterm infants with bronchiolitis (p = 0.03). *p<0.05. Total neutrophil TLR4 in BAL fluid was significantly greater (p<0.05) than in blood for all three groups (not shown).

All neutrophils in the blood and BAL fluid were positive for expression of TLRs 2, 4, 7, 8 and 9. There was no difference between cell surface TLR2 expression on neutrophils from blood or BAL fluid or between preterm, term or control infants (fig 2A, B), nor was there any difference between groups or compartments for total TLR2 (fig 2A, C). There was no difference in neutrophil cell surface TLR4 expression between the three groups in either the blood or the BAL fluid (fig 3A, B). However, in blood neutrophils the total TLR4 for term infants with RSV bronchiolitis was significantly less than for preterm infants with RSV bronchiolitis (0.51 (0.023) vs 0.70 (0.038), p = 0.036), and both were less than for control infants (1.0 (0.035), p = 0.005; fig 3B).

In the BAL fluid, total neutrophil TLR4 was significantly less in term infants with RSV bronchiolitis than in preterm infants with RSV bronchiolitis (0.70 (0.02) vs 0.92 (0.02), p = 0.03), and both were less than in control infants (1.70 (0.028), p = 0.001; fig 3A, C). For each patient group there was significantly more total neutrophil TLR4 expressed in the BAL fluid than in the blood (p<0.05).

For the TLRs expressed intracellularly (TLRs 7, 8 and 9) there was considerably less overall expression in neutrophils from blood and BAL fluid than those expressed on the cell surface (TLRs 2 and 4). The total ABC range for TLRs 7, 8 and 9 was 982–9070 compared with the much higher range of total TLR2 and TLR4 of 0.138–1.7×10^6^. There were no significant differences in neutrophil expression of the intracellular TLRs 7, 8 and 9 between term and preterm infants with bronchiolitis and controls in either blood or BAL fluid neutrophils or between BAL fluid and blood within each group (data not shown).

### Neutrophil expression of TLR mRNA in infants with severe RSV bronchiolitis compared with healthy infants

**Figure 4:**
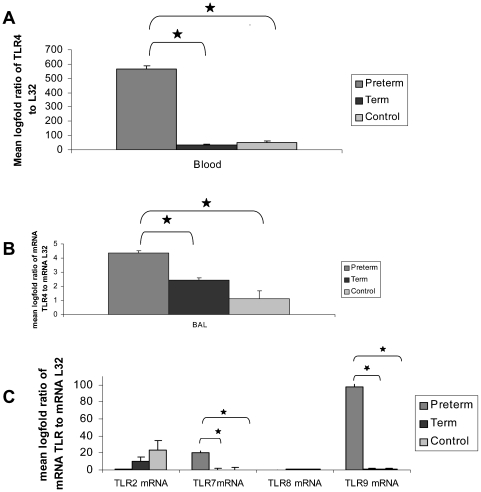
Neutrophil TLR4 mRNA expression predominantly occurs in the blood compared with the bronchoalveolar lavage (BAL) fluid and is similar in term and control infants. Data presented as mean (SEM) logfold ratio after expression of mRNA assessed by RT-PCR for all Toll-like receptors (TLRs) standardised as a ratio to mRNA for the housekeeping gene L32. (A) Neutrophil TLR4 mRNA was significantly increased in blood neutrophils, mRNA expression was greater in preterm infants (n = 16) than in term infants with respiratory syncytial virus (RSV) bronchiolitis (n = 15), but there was no significant difference between term infants with RSV bronchiolitis and controls (n = 8). (B) Neutrophil TLR4 mRNA was significantly increased in BAL fluid neutrophils from preterm infants with RSV bronchiolitis (n = 16) compared with term infants with RSV bronchiolitis (n = 15), which was similar to control infants (n = 8). There was considerably less mRNA TLR4 for each patient group in the BAL fluid than in the blood (not shown). (C) As the blood seemed to be the main site of TLR4 mRNA production, neutrophil mRNA expression of TLR 2, 7, 8 and 9 was determined for blood neutrophils for preterm (n = 16) and term infants with RSV bronchiolitis (n = 15) and for controls (n = 8). There was no significant difference in the expression of blood neutrophil mRNA TLR2 between the three patient groups. Preterm infants had significantly greater expression of TLR7 and 9 in blood neutrophils than term or control infants (p<0.05). The low levels of expression of neutrophil TLR8 in term infants with RSV bronchiolitis were similar to the levels in control infants; levels of TLR8 in preterm infants with RSV bronchiolitis were undetectable. *p<0.05.

In the blood, neutrophil mRNA expression for TLR4 was similar for term infants with RSV bronchiolitis and control infants (34.84 (2.168) and 48.16 (2.758), respectively, p = 0.625; data expressed as mean (SEM) logfold ratio of TLR mRNA to L32 mRNA). Blood neutrophil TLR4 mRNA expression of preterm infants with RSV bronchiolitis (566.27 (19.825)) was much greater than that of term infants with RSV bronchiolitis (p = 0.005) or controls (p = 0.021; fig 4A).

In the BAL fluid, neutrophil TLR4 mRNA expression was significantly less than in the blood for each patient group. Similarly, BAL fluid neutrophil mRNA expression for TLR4 was greater for preterm infants with RSV bronchiolitis (4.38 (0.122)) than for term infants with RSV bronchiolitis (2.46 (0.129), p = 0.037) and for controls (1.12 (0.577), p = 0.034). As in the blood, BAL fluid neutrophil TLR4 mRNA expression for term infants with RSV bronchiolitis was similar to that of controls (p = 0.6, fig 4B).

To determine if the increased expression of TLR4 mRNA in the blood compared with the BAL fluid was also observed for the other TLRs, the mean mRNA expression of TLRs 2, 7, 8 and 9 in blood neutrophils for the two groups with RSV bronchiolitis and the control group was measured (fig 4C). For TLR7 and 9 there was a similar pattern of greatly increased neutrophil mRNA expression in preterm infants with RSV bronchiolitis compared with the other two groups. Neutrophil TLR7 mRNA was undetectable in controls. There were only low and similar levels of expression of neutrophil TLR2 in the three groups and of TLR8 mRNA in term infants with RSV bronchiolitis and controls (p>0.05). There was no detectable expression of neutrophil TLR8 mRNA in preterm infants with RSV bronchiolitis. In the BAL fluid there was very little neutrophil mRNA expression of TLRs 2, 7, 8 and 9 compared with blood (data not shown).

## Discussion

This study has shown for the first time that neutrophils from infants with severe RSV bronchiolitis are activated both in the peripheral circulation and, to a greater degree, in the lower airways. This was associated with reduced neutrophil TLR4 in both compartments, with the lowest amounts for term infants with RSV bronchiolitis. The expression of neutrophil TLRs 2, 7, 8 and 9 in both blood and BAL fluid was similar in all three groups. This finding of reduced TLR4 protein expression raises the possibility of an impaired innate immune response and contrasts with findings of many clinical studies which have shown highly overexuberant inflammatory responses during RSV bronchiolitis. This is the first study to investigate the role of TLRs in both the airway and circulation of infants with RSV bronchiolitis. We studied the expression of the human TLRs currently believed to be involved in viral recognition in a group of infants with the most severe manifestations of RSV disease and no known risk factors. We analysed infant neutrophils, the biology and immune function of which is very different from neutrophils of adults.^29^

This work has some limitations. First, as only infants with severe disease were intubated, we were unable to collect BAL fluid samples from infants with mild or moderate disease to determine whether these changes were more marked with increasing disease severity as suggested by genetic susceptibility studies.^25^ Second, although our findings may suggest a constitutive reduction in neutrophil TLR4 expression in some infants, we do not know whether expression was altered before the onset of RSV disease or after recovery. RSV F protein has been shown to bind to TLR4 on monocytes,^30^ and we have some preliminary data which suggest that the RSV virus may bind to, or associate with, neutrophils in the BAL fluid of RSV-infected infants. Whether this results from an interaction between RSV and a specific receptor such as TLR4 has not been determined. We have undertaken some preliminary in vitro work with neutrophils from healthy adults which shows no evidence of interference by RSV with TLR4 antibody binding or TLR4 detection in our assay. Additional evidence to suggest that RSV F protein binding is not interfering with TLR4 detection is provided by the observation that TLR4 expression on the surface of neutrophils was similar between our three groups and between the blood and the BAL fluid within the groups, whereas there are differences for these comparisons in total TLR4 expression.

As the neutrophils from the peripheral circulation of infants with severe RSV bronchiolitis expressed higher levels of CD11b than uninfected controls, although recirculation from the lung cannot be excluded, it appears that they become partially activated or primed in the peripheral circulation before recruitment into the lung. In the lung, neutrophils express higher levels of total and cell surface CD11b and MPO, which suggests either progressive activation^31^ ^32^ ^33^ and release of intracellular MPO by degranulation at this site or, alternatively, the selective recruitment of highly activated neutrophils from the peripheral circulation.

The observed decrease in neutrophil intracellular TLR4 protein expression could be due to reduced synthesis, mobilisation to the cell surface or increased degradation. The first two of these seem unlikely as, compared with controls, neutrophil TLR4 mRNA expression was greatly increased in preterm and similar in term infants and TLR4 expression on the surface of neutrophils was similar between the three groups. A precedent for TLR4 degradation comes from epithelial cells where TLR4/MD2 complexes are degraded once engaged by an agonist.^34^ The third explanation alone seems unlikely as neutrophil TLR4 was not only reduced in BAL fluid neutrophils—where RSV is present in large titres—but also on blood neutrophils. There is currently no evidence to suggest that RSV causes a viraemia.^7^

As only the expression of TLR4 and not of other TLRs is markedly reduced in the main infiltrating inflammatory cell during active infection, this suggests both a specific effect on TLR4 expression and that neutrophils and TLR4 play an important role in host defence against RSV infection. TLR4 signalling has primarily been studied in airway epithelial cells during RSV infection. There is very little work on RSV infection and TLR pathways in blood-derived cells and none, to our knowledge, in clinical studies that investigated neutrophils. Deficiencies in TLR pathways have been shown to be important in other viral infections and modulate host defence—for example, the *Vaccinia* virus protein A46R blocks TLR4 signalling^35^ in human epithelial tumour cell lines (HeLA cells) and *Vaccinia* virus lacking A46R has attenuated virulence.

One of the striking findings of our study was that, in neutrophils of preterm infants with RSV bronchiolitis compared with controls, despite reduced protein expression of neutrophil TLR4 in the blood and BAL fluid, there were vastly increased levels of TLR4 mRNA in the blood. In preterm infants this may represent immaturity of the TLR4 transcription pathway. However, in previous unpublished studies we have measured mRNA for TLRs 2, 4, 7 and 9 in two groups of controls (those born at term and those born preterm) and found no differences in expression between the groups. In the preterm infants this may represent an appropriate response to RSV infection with increased degradation and utilisation of TLR4-dependent pathways to help clear the infection, or these findings may reflect the immunological immaturity of these infants. As the expression of TLRs has been shown to increase with gestational age,^21^ the finding that total neutrophil TLR4 is much less in term infants than in preterm infants with severe RSV disease may suggest that term infants with severe disease have a constitutive abnormality in TLR4 expression. This may be related to the genetic polymorphisms in TLR4 that are associated with an increased risk of severe disease^24^ ^25^ and with impaired TLR4 signalling in response to RSV F protein.^27^

This study has highlighted important aspects about the role of neutrophils in RSV disease and the differences in the immunopathogenesis in preterm and term infants. In vitro studies are needed to determine whether the abnormalities in TLR4 expression contribute to or are a result of RSV disease. TLR4 agonists are currently undergoing phase II clinical trials, so elucidating these mechanisms further will have important therapeutic implications for infants with severe RSV bronchiolitis.
